# Transcriptomics Provides a Genetic Signature of Vineyard Site and Offers Insight into Vintage-Independent Inoculated Fermentation Outcomes

**DOI:** 10.1128/mSystems.00033-21

**Published:** 2021-04-13

**Authors:** Taylor Reiter, Rachel Montpetit, Shelby Byer, Isadora Frias, Esmeralda Leon, Robert Viano, Michael Mcloughlin, Thomas Halligan, Desmon Hernandez, Rosa Figueroa-Balderas, Dario Cantu, Kerri Steenwerth, Ron Runnebaum, Ben Montpetit

**Affiliations:** a Food Science Graduate Group, University of California Davis, Davis, California, USA; b Department of Viticulture and Enology, University of California Davis, Davis, California, USA; c Department of Population Health and Reproduction, University of California, Davis, California, USA; d Department of Chemical Engineering, University of California, Davis, California, USA; e Crops Pathology and Genetics Research Unit, USDA Agricultural Research Service, Davis, California, USA; University of Naples Federico II

**Keywords:** *Saccharomyces cerevisiae*, fermentation, gene expression, microbiome, transcriptomics

## Abstract

Ribosomal DNA amplicon sequencing of grape musts has demonstrated that microorganisms occur nonrandomly and are associated with the vineyard of origin, suggesting a role for the vineyard, grape, and wine microbiome in shaping wine fermentation outcomes. Here, ribosomal DNA amplicon sequencing from grape musts and RNA sequencing of eukaryotic transcripts from primary fermentations inoculated with the wine yeast Saccharomyces cerevisiae RC212 were used to profile fermentations from 15 vineyards in California and Oregon across two vintages. These data demonstrate that the relative abundance of fungal organisms detected by ribosomal DNA amplicon sequencing correlated with neither transcript abundance from those same organisms within the RNA sequencing data nor gene expression of the inoculated RC212 yeast strain. These data suggest that the majority of the fungi detected in must by ribosomal DNA amplicon sequencing were not active during the primary stage of these inoculated fermentations and were not a major factor in determining RC212 gene expression. However, unique genetic signatures were detected within the ribosomal DNA amplicon and eukaryotic transcriptomic sequencing that were predictive of vineyard site and region. These signatures included S. cerevisiae gene expression patterns linked to nitrogen, sulfur, and thiamine metabolism. These genetic signatures of site offer insight into specific environmental factors to consider with respect to fermentation outcomes and vineyard site and regional wine characteristics.

**IMPORTANCE** The wine industry generates billions of dollars of revenue annually, and economic productivity is in part associated with regional distinctiveness of wine sensory attributes. Microorganisms associated with grapes and wineries are influenced by region of origin, and given that some microorganisms play a role in fermentation, it is thought that microbes may contribute to the regional distinctiveness of wine. In this work, as in previous studies, it is demonstrated that specific bacteria and fungi are associated with individual wine regions and vineyard sites. However, this work further shows that their presence is not associated with detectable fungal gene expression during the primary fermentation or the expression of specific genes by the inoculate Saccharomyces cerevisiae strain RC212. The detected RC212 gene expression signatures associated with region and vineyard site also allowed the identification of flavor-associated metabolic processes and environmental factors that could impact primary fermentation outcomes. These data offer novel insights into the complexities and subtleties of vineyard-specific inoculated wine fermentation and starting points for future investigations into factors that contribute to regional wine distinctiveness.

## INTRODUCTION

During vinification, grape musts are transformed to wine through microbial metabolism, including fermentation of grape sugars into alcohols. In both inoculated and spontaneous fermentations, Saccharomyces cerevisiae often becomes the dominant fermentative organism due to a number of adaptations that support the rapid consumption of sugars and production of ethanol (1). However, complex microbial communities consisting of other eukaryotic microorganisms and bacteria are present and active and make significant contributions to the wine-making process and final product (2–6). Referred to collectively as non-*Saccharomyces* organisms, these microbes often originate from the vineyard or the winery itself (7, 8). In recognition of the important role these microbes have in wine making, selected non-*Saccharomyces* yeasts are increasingly being inoculated into commercial fermentations to impart beneficial properties (e.g., bioprotection, lower ethanol, or distinct sensory characteristics) (9). Grape must and wine treatments with sulfur dioxide (SO_2_) are also commonly used to control microbial populations, including spoilage organisms, but many microorganisms survive SO_2_ treatment and contribute to fermentation and wine chemistry outcomes (6, 10, 11).

The persistence of vineyard- and winery-derived microorganisms throughout the wine-making process, as well as the potential for these organisms to influence grape berry development prior to harvest, has led to the idea that certain microorganisms unique to a region or vineyard may contribute to region-specific wine characteristics (12–14). In support of a role of microbial biogeography in regional wine characteristics, microorganisms in vineyards, wineries, and grape musts are known to be associated with their region of origin (4, 7, 8, 15–22). Moreover, the abundance of some organisms in grape must correlates with metabolite concentrations in finished wine, further associating microbial biogeography with fermentation outcomes and wine quality (16, 23). Still, relatively little is known about how the majority of non-*Saccharomyces* microorganisms present in must impact wine fermentation outcomes, but an increasing number of studies are tackling this complex problem (24, 25). Recent studies have documented increased glycerol accumulation and aroma profiles using sequential inoculation or coinoculation of S. cerevisiae with a single non-*Saccharomyces* yeast species under enological conditions (26–35). While outcomes are diverse, which may be expected given the variety of starting must and culture conditions used across studies, many report consistent alterations in wine, such as a higher glycerol content from fermentations inoculated with S. cerevisiae and Starmerella bacillaris (30, 31, 35).

How these altered fermentation outcomes occur remains a difficult question to address, as a given outcome may be the direct result of metabolism by the non-*Saccharomyces* organism or the result of the organism altering S. cerevisiae metabolism via direct or indirect interactions (36–38). In support of the latter, the presence of non-*Saccharomyces* organisms has been shown to increase the rate and diversity of resource uptake by S. cerevisiae in early fermentation (37–39). In controlled steady-state bioreactor fermentations, the presence of Lachancea thermotolerans was found to increase the expression of S. cerevisiae genes important for iron and copper acquisition (40). Such interactions are not limited to fungi, as lactic acid bacteria can induce epigenetic changes (e.g., [*GAR+*] prion) in S. cerevisiae that alter glucose metabolism (41–43). Such abilities of non-*Saccharomyces* organisms to impact S. cerevisiae metabolism and fermentation outcomes raise the question of whether the microbial biogeography of vineyard sites persists in fermentations, thereby influencing wine outcomes in a site-specific manner. In addition, microbial diversity changes as the primary fermentation progresses and S. cerevisiae becomes dominant (44), suggesting that a changing microbial community could provide feedback to impact fermentation progression in multiple distinct ways. Currently, relatively little is known about these interspecies interactions and how this influences S. cerevisiae, which as a field must be addressed to understand how microbial community dynamics impact wine fermentation outcomes, chemistry, and sensory characteristics.

Past inquiries into the microbial communities of grape must and wine related to regional distinctiveness have focused on assaying the presence of specific microbes based on ribosomal DNA amplicon sequencing (4, 8, 15–21, 45). DNA sequencing has the advantages of capturing both metabolically active and inactive organisms, due to the relative stability of the DNA molecule, offering evidence of a rich history of the microbial community prior to sampling. Ribosomal DNA amplicon data further provide a measure of which microbes may be active at the time of sampling or may become active in the future. While microbiome DNA sequencing of grape musts supports regionally distinct microbial signatures, the identity of microbes other than S. cerevisiae that metabolically contribute to the primary alcoholic fermentation remains largely unknown. This information is critical when considering the possibility that a particular microbe influences wine fermentation outcomes via metabolism or interspecies interactions.

One measure of metabolic activity that is relatively accessible and can be applied at scale to address this issue is the measurement of gene expression in both S. cerevisiae and non-*Saccharomyces* organisms. An interrogation of the genes that are “on” at a given time using RNA sequencing provides important information about the activities an organism may perform. In addition, the RNA molecule assessed by transcriptomics is constantly turned over within cells and is relatively unstable compared to DNA, which makes transcriptomics a good indicator of microbial activity and viability at the time of sampling. For example, early in fermentation, S. cerevisiae turns on genes required for glucose metabolism and represses expression of genes needed for the metabolism of other carbon sources, a pattern that reverses toward the end of fermentation, when glucose is depleted and S. cerevisiae must find alternative energy sources (46). These patterns of gene expression are readily observed using transcriptomics (46, 47), which is increasingly being applied to understand wine fermentation outcomes (37–40, 48).

Here, microbial populations present in Pinot noir musts from California and Oregon were characterized in multiple vintages using ribosomal DNA amplicon data from grape must samples prior to inoculation. In addition, eukaryotic gene expression data were generated across subsequent fermentation time points. Within these data, genetic signatures (i.e., DNA and RNA profiles) of vineyard site and region can be discerned, with total precipitation during the growing season being one vineyard-associated factor identified to correlate with site-specific genetic signatures. While DNA profiles reliably predict both vineyard site and region, these profiles did not correlate with the RNA profiles of the primary fermentations, including gene expression of the inoculated S. cerevisiae RC212 strain. These findings suggest that other characteristics of the must influenced S. cerevisiae RC212 strain gene expression more than the grape must microbiome, as measured by ribosomal DNA amplicon sequencing. A comparison of DNA sequencing and gene expression data also indicates that the majority of organisms detected by ribosomal DNA sequencing hours prior to inoculation lack detectable gene expression following inoculation, thus lowering the likelihood that many of these organisms significantly impact fermentation outcomes during the primary stage of fermentation. Finally, using S. cerevisiae RC212 gene expression patterns and the associated functions of the genes identified, it was possible to identify flavor-associated metabolic processes and environmental factors that may contribute to vineyard-specific fermentation outcomes.

## RESULTS AND DISCUSSION

To investigate the influence of vineyard site on wine fermentation outcomes across multiple vintages, standardized fermentations of Pinot noir were performed using grapes from 15 vineyard sites in California and Oregon (see Fig. S1A in the supplemental material). As part of a larger study (49–51), in 2016, 2017, and 2019, microbiome samples for DNA isolation and ribosomal DNA amplicon sequencing were taken approximately 2 to 3 h prior to inoculation from four independent fermentations per vineyard site. In the 2017 and 2019 vintages, two primary fermentations from each site were also profiled using RNA sequencing approaches to perform eukaryotic gene expression analyses at multiple fermentation time points after inoculation with the wine yeast RC212. All grape processing and temperature-controlled fermentations were performed at the UC Davis Teaching & Research Winery to standardize vinification and minimize contributions from other factors (e.g., winery and winemaker) to the microbiome and transcriptome.

10.1128/mSystems.00033-21.1 FIG S1 Diversity of vineyards and ribosomal DNA profiles in this study. (A) Map displaying the 15 vineyard locations across eight American viticultural areas (AVA) in California and Oregon. (B and C) Bacterial (B) and fungal (C) ribosomal DNA amplicon sequencing Chao 1 and Shannon alpha diversity for mean species diversity per vineyard site, averaged across vintages. Download FIG S1, PDF file, 0.3 MB. Copyright © 2021 Reiter et al. 2021 Reiter et al. https://creativecommons.org/licenses/by/4.0/ This content is distributed under the terms of the Creative Commons Attribution 4.0 International license.

### DNA abundance by ribosomal amplicon sequencing is a poor predictor of detectable gene expression during fermentation.

**FIG 1:**
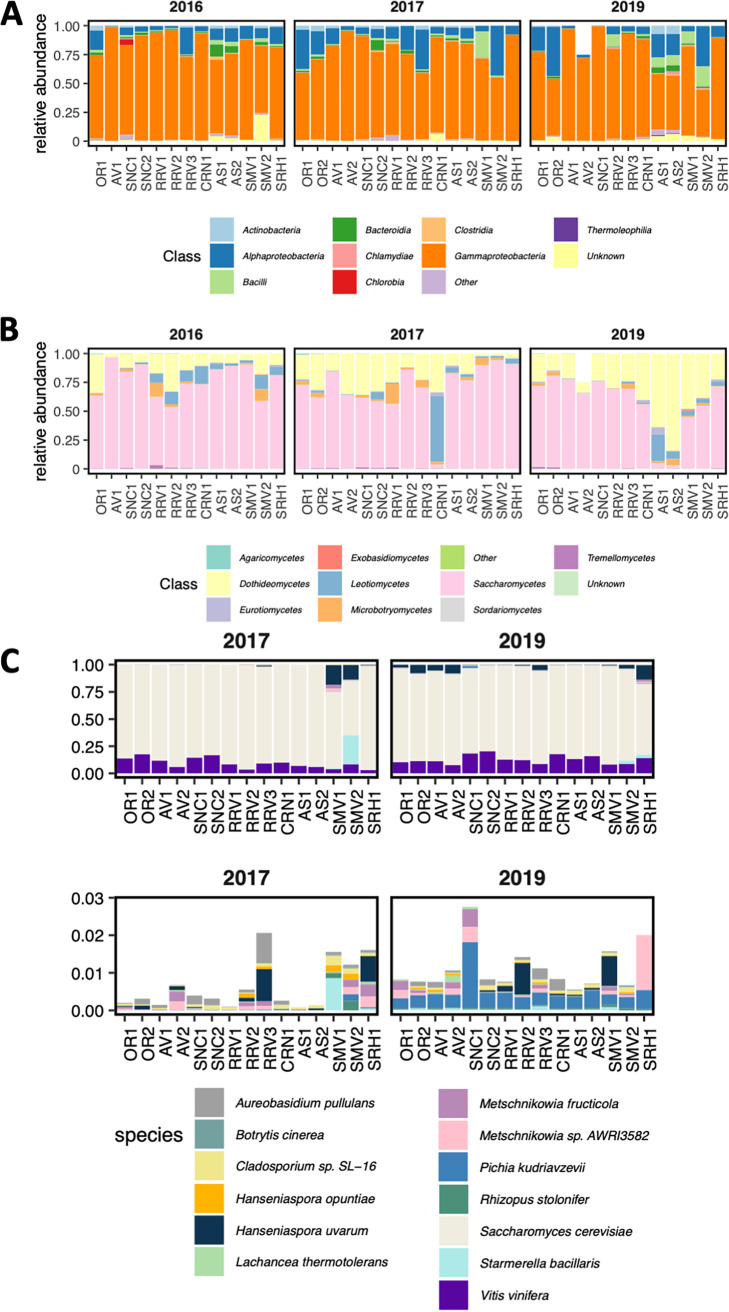
Microbial diversity in grape must and fermentation microbiomes from different vineyard sites. (A and B) Relative abundance of taxonomic ranks in ribosomal DNA amplicon sequencing data capturing bacteria (A) and fungi (B). Samples taken from fermentations from the same vineyard site and vintage are combined and reflect relative abundance of organisms from four fermentation tanks. Only three tanks were fermented for AV2 in 2019 due to a smaller harvest. (C) Relative abundance of all genes expressed by a detected organism during fermentation from the 2017 and 2019 vintages. (Top) All organisms; (bottom) organisms that account for less than 3% of mapped reads in each sample. Only organisms present in more than one fermentation are plotted.

When ribosomal DNA amplicon sequencing of bacteria and fungi was carried out, 3,254 distinct bacterial sequences and 2,452 distinct fungal sequences were detected in grape must samples (Fig. 1A and B), with a greater mean species diversity per vineyard site for bacteria than for fungi (Fig. S1B). However, the core microbiome—i.e., the species present in 90% of all grape musts across all vintages with at least 1% abundance—was larger for fungi than bacteria. The core microbiome consisted of 11 bacterial variants classified to nine taxonomic ranks and 19 fungal variants classified to 10 taxonomic ranks. All bacteria in the core microbiome belonged to the phylum *Proteobacteria* and were dominated by the genus *Tatumella* (Fig. S2). *Tatumella* has previously been identified as a dominant genus in other red wine fermentations, where it correlated with total acid (by titration) in grape must (52). Three of the most abundant bacterial sequence variants were identified as belonging to the acetic acid-producing genus *Gluconobacter* (Fig. S2). *Gluconobacter* is one of three genera of acetic acid bacteria associated with wine spoilage and the only genus identified among dominant organisms (53). *Gluconobacter* spp. are primarily active in grape must, as the wine environment restricts growth of organisms in this genus (53). Fungi in the core microbiome belonged to a single phylum, *Ascomycota*, with all fermentations dominated by the genus *Hanseniaspora*, in particular Hanseniaspora uvarum. *H. uvarum* cannot complete alcoholic fermentation alone, but it participates in and can alter the quality outcomes of wine fermentations (54). The fungal genus *Botrytis* was also identified among dominant organisms (Fig. S2), but these analyses lacked the ability to resolve whether the particular organisms detected belonged to the spoilage organism Botrytis cinerea or another species in the genus *Botrytis*. Through this work, must microbiome sequencing was extended to include the 2019 vintage, with results largely matching findings from previous vintages across these same vineyard sites (51). The observed microbial community composition was consistent with organisms previously shown to be present at the initial stages of the wine-making process (4, 16–18, 52).

10.1128/mSystems.00033-21.2 FIG S2 Some ribosomal sequencing variants were detected across vineyards and vintages. Top 20 most abundant ribosomal DNA amplicon sequencing variants across vintages. Labelled as genus or the next lowest taxonomic rank of classification. (A) Bacteria; (B) fungi. *Tatumella* was the most abundant bacterial amplicon sequencing variant across vineyards and vintages, while *Hanseniaspora* was the most abundant fungal amplicon sequencing variant. Download FIG S2, PDF file, 0.1 MB. Copyright © 2021 Reiter et al. 2021 Reiter et al. https://creativecommons.org/licenses/by/4.0/ This content is distributed under the terms of the Creative Commons Attribution 4.0 International license.

Ribosomal DNA amplicon sequencing is expected to capture cells that are metabolically active, inactive, or dead due to the stability of the DNA molecule. In contrast, gene expression profiling via RNA sequencing is expected to be biased toward living cells. Moreover, the identity of the gene transcripts present at the time of sampling further provides information about what metabolic activities the cell may be performing. While traditional RNA sequencing produces sequencing reads from an entire transcript, 3′-tag RNA sequencing (3′ Tag-seq) was employed in this work, which produces one molecule per transcript by sequencing approximately 100 bp upstream of the 3′ end of a sequence (55). This sequencing chemistry requires a poly(A) tail, limiting the sequenced fraction of the transcriptome almost entirely to polyadenylated eukaryotic mRNAs. Using 3′ Tag-seq, eukaryotic gene expression was profiled during fermentation using samples taken at multiple time points after inoculation (i.e., 16, 64, and 112 h in 2017 and 2019, plus 2 and 6 h postinoculation in 2019). The selected sampling times included time points in early fermentation, mid-fermentation, and late fermentation based on Brix values (Table 1).

**TABLE 1:** Average Brix values across fermentations at time of RNA-seq sampling in the 2017 and 2019 vintages

Sampling time (h)^*a*^	Avg (SD) °Bx in vintage	
	2017	2019
2	NA^*b*^	24.3 (0.71)
6	NA	24.3 (0.74)
16	22.6 (1.86)	23.0 (0.72)
64	6.58 (2.72)	6.26 (2.11)
112	−0.32 (1.25)	−0.83 (0.56)

a Hours after inoculation.

b NA, not available.

From the resulting 3′ Tag-seq data, it was observed that relatively few eukaryotic microbes were detected during these Pinot noir fermentations (Fig. 1C). Considering all 15 sites together, only 18 eukaryotic species were detected. As expected for an inoculated fermentation, S. cerevisiae transcripts accounted for the majority of sequences across all fermentations at all time points. To assess whether noninoculated S. cerevisiae strains were responsible for some fraction of sequence reads, the transcriptome was compared against all annotated S. cerevisiae genomes in GenBank, as well as a genome assembly of S. cerevisiae RC212. While non-RC212 S. cerevisiae strains were detectable in every fermentation, this fraction accounted for less than 1% of uniquely identifiable sequences. In all fermentations, Vitis vinifera transcripts were also identified (Fig. 1C). The detection of non-RC212 S. cerevisiae, Vitis vinifera, and other fungal organisms also indicates that the sequencing depth obtained was sufficient to detect RNA from organisms other than the inoculated and dominant RC212 yeast.

**FIG 2:**
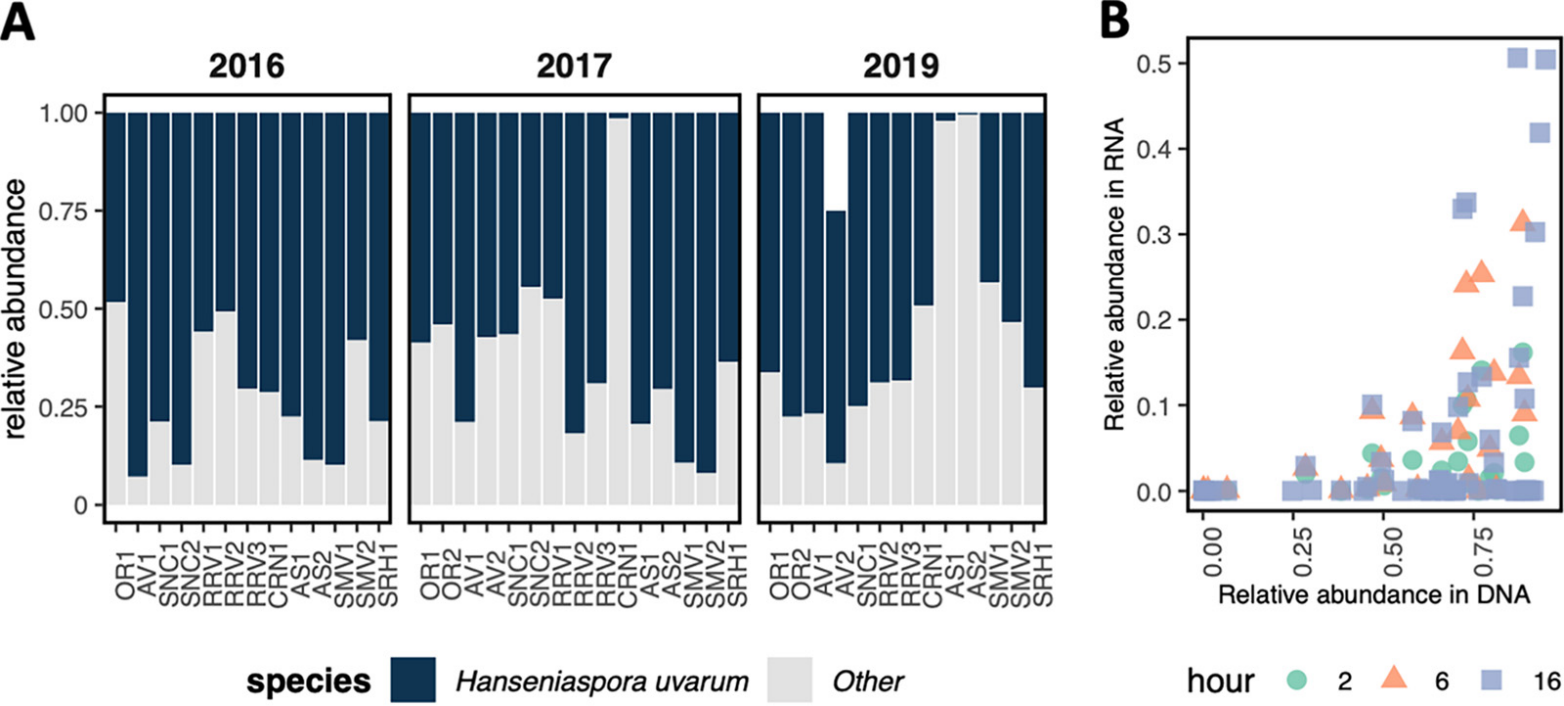
*H. uvarum* ribosomal DNA amplicon sequencing data does not strongly correlate with relative abundance in RNA sequencing data. (A) Bar chart of relative abundance of *H. uvarum* compared to other non-*Saccharomyces* species across fermentations from each site based on amplicon sequencing data of ribosomal DNA. (B) Scatterplot of relative abundance of *H. uvarum* as determined by amplicon sequencing of ribosomal DNA (*x* axis) versus RNA sequencing (*y* axis). Points are colored by number of hours after inoculation that RNA sequencing samples were sampled from the fermentations.

In comparing organisms detected via DNA sequencing and 3′ Tag-seq RNA sequencing, only four (Aureobasidium pullulans, H. uvarum, Hanseniaspora vineae, and S. cerevisiae) of 397 distinct fungal species definitively identified by ribosomal DNA profiling were detected using gene expression data. This was unchanged in the 2019 transcriptome profiling samples taken at 2 and 6 h after inoculation. These data suggest that organisms detected by amplicon sequencing ∼2 to 3 h prior to inoculation were not highly active postinoculation, even well before S. cerevisiae would begin to produce inhibitory concentrations of ethanol based on Brix values (Table 1). Ribosomal DNA sequencing data indicated that of the four organisms detected by both sequencing methods, *H. uvarum* was highly abundant in all musts from all vineyard sites prior to inoculation (Fig. 2A). Still, the relative abundance of *H. uvarum* in grape must from ribosomal DNA amplicon sequencing was only weakly correlated with relative abundance of RNA from the fermentation samples taken at 2, 4, and 16 h (2 h, *R*^2^ = 0.21, *P* < 0.05; 6 h, *R*^2^ = 0.28, *P* < 0.01; 16 h, *R*^2^ = 0.14, *P* < 0.01). Moreover, while these values are weakly correlated, *H. uvarum* had almost no detectable gene expression in fermentations from many sites where it dominated the DNA profile of the grape must just prior to inoculation (Fig. 2B). In the case of *A. pullulans*, DNA in grape must was not correlated with gene expression during fermentation (2 h, *R*^2^ = −0.03, *P* = 0.60; 6 h, *R*^2^ = −0.025, *P* = 0.53; 16 h, *R*^2^ = 0.10, *P* < 0.05). These results indicate that most of the identified eukaryotic microorganisms in grape must by DNA profiling likely have little metabolic activity in these inoculated fermentations even when the organisms are detected at high abundance and are detectable via both sequencing methods.

It is important to consider if a lack of detectable gene expression for non-*Saccharomyces* fungal species could be reflective of a technical issue or have a biological cause. This is considered unlikely, since both DNA and RNA sequencing require similar protocols for extraction of nucleic acids from cells that should perform approximately equally across samples. Moreover, the RNA sequencing performed here relies on highly conserved biological processes (e.g., mRNA polyadenylation); hence, while RNA sequencing could have failed for one or a few organisms, it should not fail across many fermentations for the large majority of organisms seen in this work. Moreover, of the 16 non-*Saccharomyces* fungi detected via RNA sequencing, eight of these organisms were not detected at the genus level by DNA profiling (*Cladosporium* sp. SL-16, *Lachancea thermotolerans*, Metschnikowia fructicola, *Metschnikowia* sp. AWRI3582, Pichia kudriavzevii, *Preussia* sp. BSL10, Rhizopus stolonifer, and Starmerella bacillaris). This suggests that transcriptomic profiling is a sensitive assay able to detect organisms present in a population that are missed by ribosomal DNA amplicon sequencing, which is likely due to an inability to resolve genus or species using ribosomal DNA sequences.

Notably, some of the organisms detected by RNA sequencing have the ability to influence fermentation outcomes: in mixed fermentations with S. cerevisiae, *S. bacillaris* has been shown to lower the final ethanol concentration and increase the concentration of glycerol (56), while *M. fructicola* increased the concentration of esters and terpenes (57). Therefore, the detection of these organisms by RNA sequencing provides valuable information with respect to the potentially active microbial population in these fermentations. These findings align well with a recent report that showed that an RNA-based sequencing strategy is a highly sensitive alternative to amplicon sequencing (58). As such, it may be appropriate to use RNA sequencing as a general method to capture the metabolically active microbial community during wine fermentation, especially when one is drawing a connection between the presence of selected organisms within the must microbiome and primary fermentation outcomes.

### Genetic signatures differentiate vineyard site, region, and vintage.

**FIG 3:**
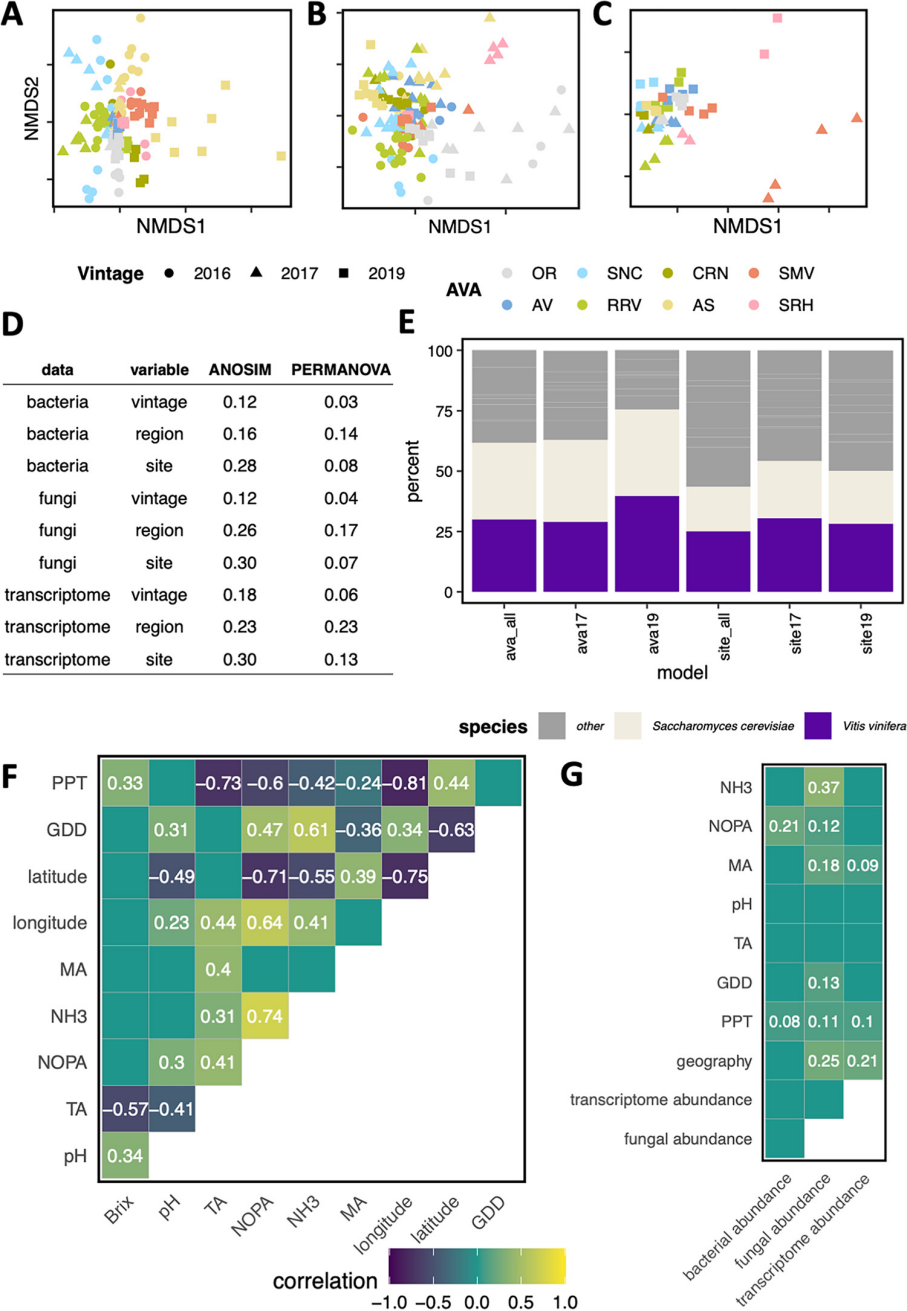
Genetic profiles correlate with vineyard, region, and vintage as well as some vineyard site and initial grape must characteristics. (A to C) Nonmetric multidimensional scaling plots of Aitchison dissimilarity of bacterial communities (A), fungal communities (B), and transcriptomes (C) across vintages. A shorter distance between two points on the graph indicates higher similarity between their genetic profiles. (D) Vineyard site, region, and vintage account for genetic diversity patterns in analysis of similarity (ANOSIM) and permutational multivariate analysis of variance (PERMANOVA). ANOSIM and PERMANOVA data are *R* values that represent strength of association, with higher *R* values indicating stronger grouping according to the parameter and statistical test. All values are significant (*P* < 0.001). (E) Percent of accuracy attributable to different organisms in random forests models. A higher percentage of variable importance was attributable to S. cerevisiae and *V. vinifera* in models that predicted region than in those that predicted vineyard site. (F and G) Correlograms representing similarities between fermentation metrics. (F) Grape must chemical parameters and vineyard site characteristics were correlated in the 2017 and 2019 vintages. Squares are labeled with correlation values from Pearson’s correlation. Only comparisons with *P* values of <0.05 are displayed. (G) Bacterial, fungal, and transcriptome profiles correlated with some vineyard site and grape must chemical characteristics. Squares are labeled with correlation values from Mantel tests. Only comparisons with an FDR of <0.1 are displayed. PPT, precipitation; GDD, growing degree days; MA, malic acid; TA, titratable acidity.

The region and site from which grapes are harvested can have an important influence on the character of a resulting wine based on a variety of factors (e.g., climate, soil type, vine nutrition, grape-associated microbes, etc.). As such, the data generated using DNA and RNA sequencing strategies during these Pinot noir fermentations may be reflective of vineyard site through the generation of unique genetic signatures. To investigate this concept, DNA and RNA sequencing samples were grouped by vineyard site, region, and vintage to see if there were detectable differences among these groups. Using analysis of similarities (ANOSIM) and permutational multivariate analysis of variance (PERMANOVA; see Materials and Methods), it was determined that all three factors explain differences among groups of samples, with vineyard site or region explaining the most group similarity (Fig. 3A to D). This supports the idea that fermentations have a detectable genetic signature within the DNA and RNA sequencing data that is reflective of vineyard site and region.

To understand which specific organisms and genes contribute to the genetic signatures of both vineyard site and region, machine learning classification models were built using random forests. These models weight the contribution of each feature to the predictive accuracy of the model, enabling robust identification of specific genes or organisms that differentiate vineyard sites or regions among fermentations. When data from all vintages were used in model training and testing to predict region, the models achieved 87% to 95% accuracy (Data Set S1; Fig. S3). When data from one vintage were used in model training and testing to predict region, accuracy dropped across all models but ranged from 57% to 75% (Data Set S1; Fig. S3). This suggests that models built with fermentations from all vintages better capture cross-vintage similarities, as these models select predictive variables that are consistent across the vintages studied. However, the accuracy of these models may decrease if the same set of predictive variables is not consistent in future vintages. Conversely, the accuracy of a model built from a single vintage and trained on a separate vintage will likely remain consistent across many vintages. From this, it was assumed that models trained using data from a single vintage better reflected model accuracy but that models trained using data from all vintages better reflected cross-vintage similarities. Given the goals of this study, which focused on the identification of site-specific vintage-independent factors, cross-vintage models were analyzed further.

10.1128/mSystems.00033-21.3 FIG S3 Accuracy of random forests models using bacterial and fungal ribosomal DNA profiles. Confusion matrices depicting accuracy of random forests models. Models were built with bacterial ribosomal DNA amplicon sequencing data to predict (A) vineyard site and (B) vineyard region or fungal ribosomal DNA amplicon sequencing data to predict (C) vineyard site and (D) vineyard region. The models depicted were trained on two vintages and validated on the third. Download FIG S3, PDF file, 0.07 MB. Copyright © 2021 Reiter et al. 2021 Reiter et al. https://creativecommons.org/licenses/by/4.0/ This content is distributed under the terms of the Creative Commons Attribution 4.0 International license.

10.1128/mSystems.00033-21.7 DATA SET S1 Data on the accuracy of random forests models built with fungal and bacterial ribosomal DNA amplicon sequencing data and transcriptome data. Measures of variable importance from random forests models built with the sequencing data are also provided. Download Data Set S1, XLSX file, 2.3 MB. Copyright © 2021 Reiter et al. 2021 Reiter et al. https://creativecommons.org/licenses/by/4.0/ This content is distributed under the terms of the Creative Commons Attribution 4.0 International license.

At the vineyard level, when the same data were used to generate models, predictive accuracy was on average 21.4% less than that of region-specific models (Data Set S1). However, it is important to note that this decrease in accuracy was driven by within-region classification errors for vineyards in the Willamette Valley (31-km separation), Santa Maria Valley (5-km separation), and Arroyo Seco (1-km separation) American viticultural areas (AVA) (Fig. S4). The same classification errors persisted across many models, highlighting potential within-region similarity that contributes to genetic signatures, which fits well with the concept of AVA and region-associated wine characteristics.

10.1128/mSystems.00033-21.4 FIG S4 Accuracy of random forests models using RNA sequencing. Confusion matrices depicting accuracy of random forests models built with RNA sequencing data to predict (A) vineyard site and (B) vineyard region. The models depicted were trained on one vintage and validated on the other. Download FIG S4, PDF file, 0.05 MB. Copyright © 2021 Reiter et al. 2021 Reiter et al. https://creativecommons.org/licenses/by/4.0/ This content is distributed under the terms of the Creative Commons Attribution 4.0 International license.

Across these analyses, bacterial based models outperformed, or performed as well as, fungal models for classification of site and region. This differs from previous studies, in which bacterial must samples added the least predictive power for region prediction (15), including for Pinot noir grapes grown in Australia (8). Bacterial must samples have been shown to be predictive of region in Californian Chardonnay but not Californian Cabernet Sauvignon (15), suggesting a possible cultivar-specific effect. In previous inquiries, samples were processed in vineyard-specific wineries, providing another variable that could potentially alter the measured microbiomes and the contributions attributed to bacteria and fungi.

Given that random forests models estimate the importance of each gene in determining vineyard or region classification, information from the gene expression models was used to gain insight into biological differences between vineyard sites and regions. For this, the percentage of total importance attributable to each gene and eukaryotic organism detected was calculated (Data Set S1). Vineyard-specific models weighted non-*Saccharomyces* yeast genes as a whole as most important for predictive accuracy (Fig. 3E; Fig. S5). In particular, genes from *S. bacillaris*, *M. fructicola*, *Metschnikowia* sp. AWRI3582, and *L. thermotolerans* were important for vineyard site classification. The ability of non-*Saccharomyces* gene expression to distinguish site is likely related to the unique combination of non-*Saccharomyces* organisms present in each fermentation and their infrequent detection via RNA sequencing, which results in these organisms having strong predictive power when detected. In contrast, regional models weighted S. cerevisiae and *V. vinifera* genes as having higher importance (Fig. 3E; Fig. S5). These observations may result from changes in *V. vinifera* gene expression across more diverse geographical environments, which leads to differences in the grape must and associated fermentations as detected by S. cerevisiae gene expression.

10.1128/mSystems.00033-21.5 FIG S5 Percent of accuracy attributable to different organisms in random forests models. Importance of genes expressed by different organisms in the overall model. A higher percentage of variable importance was attributable to S. cerevisiae and *V. vinifera* in models that predicted region than in those that predicted vineyard site. Download FIG S5, PDF file, 0.3 MB. Copyright © 2021 Reiter et al. 2021 Reiter et al. https://creativecommons.org/licenses/by/4.0/ This content is distributed under the terms of the Creative Commons Attribution 4.0 International license.

To more directly address how environmental factors and grape must chemistry correlate with genetic signatures, initial must chemical parameters (pH, titratable acidity, malic acid, nitrogen by *o*-phthaldialdehyde assay [NOPA], and NH_3_) and vineyard site characteristics (total precipitation, growing degree days, and geographic distance between sites) were correlated with DNA and RNA profiles using the Mantel test (see Materials and Methods). Using these analyses, geographic distance between vineyards correlated with precipitation and growing degree days, indicating that sites that are geographically closer experience more similar weather patterns, as would be expected (Fig. 3F). Among the factors tested, only precipitation correlated with all genetic profiles (Fig. 3G). Similar to geographic distance, initial chemical profiles of vineyard sites were more similar when sites were geographically closer. However, surprisingly few correlates between genetic profiles and initial grape must conditions were found (Fig. 3G). While fungal profiles correlated with initial malic acid, NOPA, and NH_3_ and bacterial profiles correlated with initial NOPA, gene expression profiles correlated only with initial malic acid levels. The finding that gene expression profiles do not correlate with initial nitrogen concentration, even though nitrogen availability is central to yeast growth and linked to the expression of hundreds of genes (46), may reflect nitrogen additions at ∼24 h after inoculation during winemaking so that all fermentations had a minimum of 250 mg/liter (see methods). Overall, the poor correlation between gene expression patterns and the factors tested suggest that other unmeasured factors are responsible for the distinctive gene expression patterns detected in these fermentations. This raises a clear need for future work that measures many factors within vineyards and fermentations to define the organism-environment interactions responsible for driving unique gene expression and cellular activities of S. cerevisiae and other microbial organisms.

### S. cerevisiae gene expression provides insight into vineyard site and region features.

S. cerevisiae is likely the best-understood eukaryote because of its use as a model system for biology, which has provided a rich set of genomic resources and databases (59). As such, S. cerevisiae gene expression can be used as a biosensor to provide insight into the fermentation environment based on the activities the yeast performs. The utility of these data is increased by the fact that S. cerevisiae gene functions are well studied in the context of wine production, S. cerevisiae is ubiquitous across all fermentations, and the transcriptomics data are dominated by reads from S. cerevisiae (e.g., data completeness). Consequently, S. cerevisiae gene expression data were queried to assess what genes, and associated functions, were important for predicting site. These data were then used to infer what aspects of the must environment may be unique from each vineyard site or region. Notably, random forests models are nondeterministic, meaning that each time a model is built, the specific genes important for predictive accuracy of that model may change, especially for genes with correlated gene expression values (60). Therefore, 100 random forests models were built for the prediction of region and vineyard site, and only S. cerevisiae genes that were shared across the majority of models were considered (Data Set S2). As discussed above, less than 1% of transcripts in any fermentation were expressed by non-RC212 S. cerevisiae, and thus the genetic signatures identified are likely specific to strain RC212.

10.1128/mSystems.00033-21.8 DATA SET S2 Measures of variable importance of genes with positive permutation variable importance values for each of 100 random forests models built with different random seeds. Download Data Set S2, XLS file, 18.9 MB. Copyright © 2021 Reiter et al. 2021 Reiter et al. https://creativecommons.org/licenses/by/4.0/ This content is distributed under the terms of the Creative Commons Attribution 4.0 International license.

From this analysis, important predictors of both site and region included flavor-associated S. cerevisiae genes involved in the formation of higher alcohols and volatile fatty acids through the Ehrlich pathway. Each site-specific and region-specific model included an average of 16 (site standard deviation [SD] = 2.9, region SD = 2.4) genes associated with flavor development in wine (Data Set S3). These genes were mostly associated with the Ehrlich pathway (site mean = 8.1 genes, SD = 2; region mean = 9 genes, SD = 1.7) and with volatile sulfur formation (site mean = 6.3 genes, SD = 1.6; region mean = 5.1 genes, SD = 1.4). Given that genes in these pathways were detectable as indicators of both region and site, variable expression of these genes could contribute to region- and vineyard-specific wine flavor profiles detected in wines from these vineyards in previous vintages (49). At this time, it remains unknown what factors within the fermentation environment cause these flavor-associated genes to differ between fermentations.

10.1128/mSystems.00033-21.9 DATA SET S3 Gene lists associated with the study. This includes genes associated with wine flavor, genes of the Com2 regulon, and genes associated with positive permutation variable importance in all 100 random forests models built with different random seeds. Download Data Set S3, XLSX file, 0.07 MB. Copyright © 2021 Reiter et al. 2021 Reiter et al. https://creativecommons.org/licenses/by/4.0/ This content is distributed under the terms of the Creative Commons Attribution 4.0 International license.

In addition to flavor-associated genes, many S. cerevisiae genes that were important for predicting both vineyard site and region are members of the Com2 regulon (Data Set S3). Expression of genes within the Com2 regulon are protective against SO_2_ stress (61). In this work, on day 2 of the cold soak ∼24 h prior to inoculation, all fermentations were adjusted to have total SO_2_ levels of 40 ppm. However, variable application of sulfur-containing fungicides in the vineyard may lead to disparate sulfur stress during fermentation and may underlie the genetic signatures of site and region that are observed. Wine strains of S. cerevisiae are more tolerant of SO_2_ than many non-*Saccharomyces* species, but SO_2_ exposure can cause inhibition of key metabolic enzymes like alcohol dehydrogenase, as well as other processes through cleavage of disulfide bonds (62, 63). Of the 511 genes dependent on Com2 activation during SO_2_ stress (61), an average of 105 genes (SD = 12.7) were important for differentiating site in our predictive models, while 101 genes (SD = 11.6) were important for predicting vineyard region. Within these gene lists are genes involved in the efflux of sulfite and bisulfite; sulfate assimilation; biosynthesis of methionine, cysteine, arginine, and lysine; and biosynthesis of the sulfur-containing vitamin biotin (Data Set S3). These pathways, and their site-specific signatures, are potential areas of future study given that sulfur metabolism can have a profound impact on the sensory attributes of a finished wine (64). In addition, while the molecular form of SO_2_ causes S. cerevisiae stress and inactivation of wine spoilage microbes (11, 61), this form is in equilibrium with the bisulfite form (HSO_3_^−^), and this ratio is dependent on wine pH (65). The bisulfite form interacts with anthocyanins and can cause color bleaching (65). This suggests that the SO_2_ stress response is a factor that would need to be considered in the context of pH and other aspects of SO_2_ wine chemistry.

In models important for predicting region only, an average of 22.4 genes per region (SD = 13.5) were predictive across all models, with an average of 14.4 genes (SD = 8.4) expressed by S. cerevisiae (Data Set S3). Interestingly, many S. cerevisiae genes that were important for predicting one region were also important for predicting other regions (*BET2*, *BET3*, *BIO4*, *EXG2*, *FAS2*, *HEM12*, *LOH1*, *MEP3*, *MRX21*, *NPT1*, *PSA1*, *SNZ3*, *THI11*, *THI13*, *THI72*, and *TUB4*), suggesting that expression of these genes differed consistently between regions. These genes encode proteins involved in diverse cellular processes, including heme biosynthesis, cell wall assembly, and synthesis and transport of fatty acids and nitrogen-containing compounds. While the underlying biochemical processes that lead to consistent expression of these S. cerevisiae genes within regions remains unknown, it was notable that *MEP3*, a gene that encodes an ammonia permease (66), was important for predicting the three regions with the lowest average initial yeast-assimilable nitrogen (Oregon, Anderson Valley, and Russian River Valley) and the region with the second highest yeast assimilable nitrogen (Santa Maria Valley) across vintages (Fig. S6). Given that nitrogen availability plays a fundamental role in fermentation outcomes (67), the ability of *MEP3* expression to identify specific regions based on nitrogen levels may be expected. It was also noted that four genes associated with thiamine availability were important for predicting multiple regions. This suggests that thiamine availability may be a factor to consider with respect to regional differences in wine fermentation outcomes, a postulate that could be measured in a future vintage. Given that gene expression is inherently noisy (68), increasing the number of site samples and the sequencing depth may improve model accuracy in the future.

10.1128/mSystems.00033-21.6 FIG S6 Initial levels of yeast assimilable nitrogen (YAN) in grape musts across vintages. Black dots mark the mean initial YAN value calculated from all fermentations in the 2017 and 2019 vintages. *MEP3*, which encodes an ammonia permease, was important for predicting the three regions with the lowest average initial YAN levels (OR, AV, and RRV) and the region with the second highest initial YAN level (SMV). Download FIG S6, PDF file, 0.05 MB. Copyright © 2021 Reiter et al. 2021 Reiter et al. https://creativecommons.org/licenses/by/4.0/ This content is distributed under the terms of the Creative Commons Attribution 4.0 International license.

Together, these results identify S. cerevisiae genes expressed during primary fermentation that are predictive of vineyard site and region in Pinot noir fermentations. Many of these genes are linked to metabolic processes that could impact wine sensory and chemistry. Consequently, these findings provide a concrete starting point for future investigation into factors that contribute to vineyard- and region-specific wine fermentation outcomes and ultimately wine chemistry and sensory characteristics.

### Conclusion.

The microbial biogeography of wine has been documented in globally distributed appellations (4, 7, 8, 15–22) and has been correlated with wine fermentation outcomes (16, 23). In inoculated cocultures, non-*Saccharomyces* microorganisms both contribute to fermentation and change the behavior of the dominant fermenter, S. cerevisiae, leading to measurable differences in wine aroma and composition (37–39). Here, it was found that grape must ribosomal DNA profiles do not correlate with detected eukaryotic gene expression patterns during primary fermentation. Given the lack of a strong correlation between fungal profiles in initial grape must and genes expressed by those organisms or the inoculated RC212 strain during primary fermentation, the use of must microbiome DNA profiles to infer contributions from these organisms to inoculated fermentation and wine sensory outcomes must be carefully considered. However, DNA profiles, in particular bacterial profiles, are predictive of vineyard site and retain signatures of site-specific processes such as total precipitation during the growing season. These profiles are rich indicators of the patterns that shape the microbial ecology of grapes and reflect differences among vineyard sites and regions, even when the same clone (e.g., Vitis vinifera L. cv. Pinot noir clone 667) is grown on each site.

Similarly, the gene expression profiles of S. cerevisiae and other eukaryotic organisms also retained signatures of vineyard site and region. Cellular functions of the S. cerevisiae genes identified as important for differentiating site included nitrogen, sulfur, and thiamine metabolism. While these processes were associated with vineyard-specific genetic signatures, few vineyard site and initial grape must chemical parameters were found to correlate with the detected fermentation transcriptome. This suggests that there are still many variables to discover that underlie the complex metabolic activities and gene expression patterns measured throughout fermentation that are linked to site. In the future, more comprehensive sequencing approaches (e.g., deeper sequencing with methods that capture the full transcriptome, more samples per site, and inclusion of bacterial gene expression) aimed at the factors and organisms identified in this work would allow a better understanding of these systems. This will need to be accompanied by measurements of many more vineyard, must, and wine characteristics to provide further predictive power and insights into the complexities and subtleties of vineyard-specific wine fermentation outcomes.

## MATERIALS AND METHODS

### Grape preparation and fermentation.

The winemaking protocol has been described previously (49, 50), but the relevant parts are reproduced with some added details below. The grapes used in this study originated from 15 vineyards in eight American viticultural areas in California and Oregon (USA). All grapes were Vitis vinifera L. cv. Pinot noir clone 667, with either rootstock 101-14 (AV1, RRV1, SNC1, SNC2, CRN1, AS1, AS2, SMV1, SMV2, and SRH1), Riparia Gloire (OR1 and OR2), or 3309C (AV2, RRV2, and RRV3). Grapes were harvested by hand at approximately 24 degrees Brix (24°Bx) and transported to the University of California, Davis, Teaching & Research Winery for fermentation. Grapes were separated into half-ton macrobins on harvest day, and Inodose (potassium metabisulfite and potassium bicarbonate) was added to achieve SO_2_ levels of 40 ppm. Upon delivery to the winery, bins were stored at 14°C until the fruit was destemmed and divided into temperature jacket-controlled tanks. N_2_ sparging of the tank headspace was performed prior to fermentation, and tanks were sealed with a rubber gasket. Grapes were cold soaked at 7°C for 3 days with SO_2_ additions made on day 2 of cold soaking to maintain a level of 40 ppm total SO_2_. On the morning of day 3, ∼20 h later, microbiome samples were taken and the musts were warmed for inoculation to 21°C with programmed pump-overs used to hold the tank at a constant temperature. Approximately 2 to 3 h after the musts reached the desired temperature, they were inoculated. For inoculation, S. cerevisiae RC212 (Lallemand) was rehydrated with Superstart Rouge (Laffort) at 0.2 g/liter and inoculated in the must at 0.25 g/liter. Fermentation progress was determined by measuring Brix values with a density meter (Anton Paar 35 DMA).

At approximately 24 h after inoculation, nitrogen content was adjusted in the fermentations by adding diammonium phosphate (DAP), according to the formula (target level of yeast assimilable nitrogen [YAN] – 35 mg/liter – initial level of YAN)/2, and Nutristart (Laffort) at 0.25 g/liter. Nitrogen was adjusted only if the target YAN level was below 250 mg/liter based on measures of ammonia and free α-amino nitrogen content (Gallery automated photometric analyzer; Thermo Fisher Scientific). Approximately 48 h after fermentation, fermentation temperatures were permitted to increase to 27°C and DAP was added as previously described. Fermentations were then continued until <0°Bx was reached. Fermentation samples were taken for Brix measurements every 12 h relative to inoculation and with RNA samples at 2 h, 6 h (2019 vintage), 16 h, 64 h, and 112 h (2017 and 2019 vintages). To ensure uniform sampling, a pump-over was performed 10 min prior to sampling of each tank. For RNA samples, 12 ml of juice was centrifuged at 4,000 rpm for 5 min, supernatant was discarded, and the cell pellet snap-frozen in liquid nitrogen. Samples were stored at −80°C until RNA extraction.

### Amplicon sequencing data processing.

DNA was extracted for amplicon sequencing and library preparation as described in references 51 and 69. The UC Davis DNA Tech Core performed sequencing using Illumina MiSeq, producing 251-bp paired-end sequences. The data were demultiplexed by barcode sequences and adapter trimmed using cutadapt (70). Taxonomically annotated amplicon sequence variant (ASV) counts were generated using DADA2 with the Silva nonredundant (NR) database (version 138) for 16S sequences and the UNITE general FASTA release (version 8.2) for internal transcribed spacer (ITS) sequences (71). All ASVs annotated as “Bacteria,Cyanobacteria,Cyanobacteriia,Chloroplast” and “Bacteria,Proteobacteria,Alphaproteobacteria,Rickettsiales,Mitochondria” were removed, as these represent plant mitochondria and chloroplast 16S sequences and not bacterial sequences. See Data Set S4 for the numbers of reads quantified in each library before and after filtering.

10.1128/mSystems.00033-21.10 DATA SET S4 Species names and accession numbers for genomes used in this study, as well as read counts from the sequencing libraries generated. Download Data Set S4, XLSX file, 0.02 MB. Copyright © 2021 Reiter et al. 2021 Reiter et al. https://creativecommons.org/licenses/by/4.0/ This content is distributed under the terms of the Creative Commons Attribution 4.0 International license.

### RNA sequencing data processing.

Yeast pellets were thawed on ice, resuspended in 5 ml Nanopure water, and centrifuged at 2,000 × *g* for 5 min, and the supernatant was aspirated. RNA was extracted using the Quick RNA fungal/bacterial miniprep kit, including DNase I column treatment (catalog no. R2014; Zymo Research). RNA was eluted in 30 μl of molecular-grade water and assessed for concentration and quality via Nanodrop spectrometry and RNA gel electrophoresis. Sample concentrations were adjusted to 200 ng/μl and used for sequencing. Single-end 3′ Tag-seq sequencing (Lexogen QuantSeq) was applied in both the 2017 and 2019 vintages, with the addition of UMI (unique molecular identifier) barcodes in 2019. The University of California, Davis, DNA Technologies Core performed all library preparation and sequencing.

The first 12 bp from each read were hard trimmed, and Illumina TruSeq adapters and poly(A) tails were removed. The program sourmash gather was used to determine the organisms present in each sample using parameters -k 31 and –scaled 2000 (72, 73). The GenBank microbial database (https://sourmash-databases.s3-us-west-2.amazonaws.com/zip/genbank-k31.sbt.zip) and eukaryotic RNA database (https://osf.io/qk5th/) were used for these queries.

Using results from sourmash, a set of reference genomes was constructed that was representative of all organisms detected within the samples. When different strains of the same species were detected, the one species detected in the largest number of samples was used as a representative species to reduce multimapping conflicts. Species present in more than two samples were included because species present in fewer than three samples would have limited predictive power. Species of the genus *Saccharomyces* other than S. cerevisiae were removed to reduce multimapping conflicts. Selected genomes were downloaded from NCBI GenBank; however, if no GTF annotation file was available for the species, the genome and GFF3 file were taken from JGI Mycocosm (74), and the GFF3 was converted to GTF using the R package rtracklayer (75). When no annotation file was available on GenBank or JGI Mycocosm, the genome of the closest species-level strain with a GTF annotation file was used.

To find closely related organisms, NCBI taxonomy was searched, selected assemblies were downloaded, and sourmash compare was used with a k size of 31 (72, 73). The organisms with the highest Jaccard similarity were considered the most similar. When no annotation file was available for similar organisms, an annotation file was generated using WebAugustus (76). See Data Set S4 for a description of the best-matched genome, the genome used for count generation, and the source of genome annotations. Reference genome FASTA files and GTF files were concatenated to generate a single reference. STAR was then used to align reads against the constructed reference with the parameters –outFilterType BySJout, –outFilterMultimapNmax 20, –alignSJoverhangMin 8, –alignSJDBoverhangMin 1, –outFilterMismatchNmax 999, –outFilterMismatchNoverLmax 0.6, –alignIntronMin 20, –alignIntronMax 1000000, –alignMatesGapMax 1000000, –outSAMattributes NH HI NM MD, –outSAMtype BAM, and SortedByCoordinate (77). For the 2019 vintage, UMI-tools was used to deduplicate alignments (78). The number of reads mapping to each gene was quantified using htseq count using the constructed reference GTF file to delineate gene regions (79). See Data Set S4 for number of reads quantified in each library.

### RC212 genome assembly and comparison.

The S. cerevisiae RC212 genome was assembled to estimate the fraction of RNA sequencing reads in each fermentation originating from non-RC212 S. cerevisiae strains. FASTQ files for accession no. SRR2967888 were downloaded from the European Nucleotide Archive (80). Reads were k-mer trimmed using the khmer trim-low-abund.py command with the parameter -k 20 (81), and the Megahit assembler was used with default parameters to assemble reads (82).

### Estimation of noninoculated yeast in RNA-seq samples.

The program sourmash gather was used to estimate the fraction of transcriptome sequencing (RNA-seq) reads (k-mers) originating from noninoculated S. cerevisiae. The sourmash gather tool estimates shared sequence similarity by comparing scaled MinHash signatures derived from k-mer profiles (72, 73). The sourmash eukaryotic RNA database (https://osf.io/qk5th/) was used, which includes all annotated S. cerevisiae genomes in GenBank (e.g., genomes that include *rna_from_genome.fna annotations), as well as our S. cerevisiae RC212 genome assembly.

### Correlation between ribosomal DNA amplicon sequencing data and 3′ Tag-seq data for non-*Saccharomyces* organisms.

Fermentations with fungal ITS amplicon sequencing data and 3′ Tag-seq were compared. First, ribosomal DNA amplicon sequencing read counts from *H. uvarum* were regressed against total 3′ Tag-seq counts from *H. uvarum* using counts from 16 h, 64 h, and 112 h of fermentation. 3′ Tag-seq counts were derived from STAR and htseq (see “RNA sequencing data processing” above). Counts were transformed into compositional counts (relative abundance) prior to linear regression (83). Linear regression was performed using the lm() function in R. This analysis was performed again separately for *H. uvarum* and *A. pullulans* using counts from the 2 h, 6 h, and 16 h samples taken in the 2019 vintage. Given that this analysis relied on reads aligned to annotated 3′ regions, a separate regression was performed a using proportion of reads assigned to a given organism derived from sourmash gather (see RNA sequencing data processing above). Only results from the first analysis were reported, as *R*^2^ values were within 0.01 between both analyses.

### ANOSIM, PERMANOVA, and NMDS.

Compositional data analysis was used for amplicon and transcriptome counts (83). The transform() function in the microbiome bioconductor package was used to transform counts by centered log ratio (84, 85). To test for differences among groups, Aitchison distance (Euclidean distance on centered log ratio [CLR]-transformed counts) was used and tested with the anosim() function (parameters: distance = “euclidean” and permutations = 9999) and the adonis2() function (parameters: method = “euclidean” and permutations = 9999) in the vegan package (86, 87). A cutoff *P* value of 0.05 was used for statistical significance. To construct nonmetric multidimensional scaling (NMDS) plots, Aitchison distance was taken using the metaMDS() function in the vegan package with the parameter distance = “euclidean.” Results were plotted using the ggplot2 package (88).

### Amplicon sequencing random forests models.

Random forests classifiers were built using the R ranger package (89). Using ASV counts produced by DADA2, counts were normalized by dividing by total number of aligned reads. The tuneRanger() function was used in the tuneRanger package to optimize each model for the parameters m.try, sample.fraction, and min.node.size (90). The ranger() function was then used to build each model with parameters from tuneRanger as well as the following: num.trees = 10000, importance = “permutation,” and local.importance = TRUE. As a supervised technique, random forests classifiers are trained on a subset of data and tested on a separate subset to calculate predictive accuracy. For models built with samples from all vintages, the createDataPartition() function in the R caret package was used to randomly but equally partition training and testing sets with a 70:30 split, ensuring that all class labels were equally represented in both sets (91). For other models, the classifier was built using all samples from two vintages and validated on the held-out vintage. Accuracy and kappa statistics were calculated for each model.

### RNA sequencing random forests model.

Counts were imported into R and normalized by dividing by total number of aligned reads (e.g., library size). Given that the random forests approach expects independent samples and that RNA sequencing was conducted in time series over the course of primary fermentation, each gene from each time series set was summarized into mean count, minimum count, maximum count, total count, and standard deviation of counts. Variable selection was performed using the vita method (92), and models were built using the same methods as with amplicon sequencing models.

To estimate vineyard- and region-specific gene importance, variable selection and model optimization were performed with 100 different seeds. For each model, gene local importance was averaged for each fermentation from a vineyard site or region in the training set and genes with positive average local permutation importance were retained. The intersection of genes from all models was then taken to determine which genes were predictive for a particular site or region in all models. Although random forests were trained on summarized gene attributes, any genes that were predictive across any attribute were retained, as these attributes were often highly correlated.

### Mantel tests.

Mantel tests were performed to assess the similarity between samples across measurements of bacterial abundance, fungal abundance, transcriptome abundance, initial grape must chemistry, and vineyard site parameters (93, 94). The Mantel test determines the correlation between the same samples in different matrices, testing whether similarities between samples estimated from one measurement type match similarities of the same samples estimated from a different measurement type (93, 94). These tests were performed using complete cases, with microbiome and transcriptome abundances from the 2017 and 2019 vintages. The vineyard site parameters total precipitation and growing degree days were estimated using the PRISM climate models including the dates April 1 to September 30 in 2017 and 2019 (95). Distance matrices were calculated for each matrix using the dist() function in R, with the parameter method = “euclidean.” with the exception of geographic distance, which was calculated using the distm() function in the package geosphere with the parameter distHaversine (ftp://sunsite2.icm.edu.pl/site/cran/web/packages/geosphere/geosphere.pdf). When distances for disparate measurement types were calculated at the same time, values were first scaled and centered using the function scale() with the parameters center = TRUE and scale = TRUE. Mantel tests were performed with the mantel() function in the vegan package with the following parameters: method = “spearman,” permutations = 9999, and na.rm = TRUE (87, 94). *P* value adjustments were applied using the function p.adjust() with the parameter method = “fdr” and a false discovery rate of a *P* value of 0.1.

### Data availability.

RNA sequencing data are available in the Sequence Read Archive under accession number PRJNA680606. Microbiome data are available under accession numbers PRJNA642839 and PRJNA682452. All analysis code is available at github.com/montpetitlab/Reiter_et_al_2020_SigofSite.
