# Supplementary figures and images for: Transcriptomics Provides a Genetic Signature of Vineyard Site and Offers Insight into Vintage-Independent Inoculated Fermentation Outcomes
Source: mSystems. 2021 Apr 13;6(2):e00033-21. doi: 10.1128/mSystems.00033-21 (PMC8546962; doi:10.1128/mSystems.00033-21)

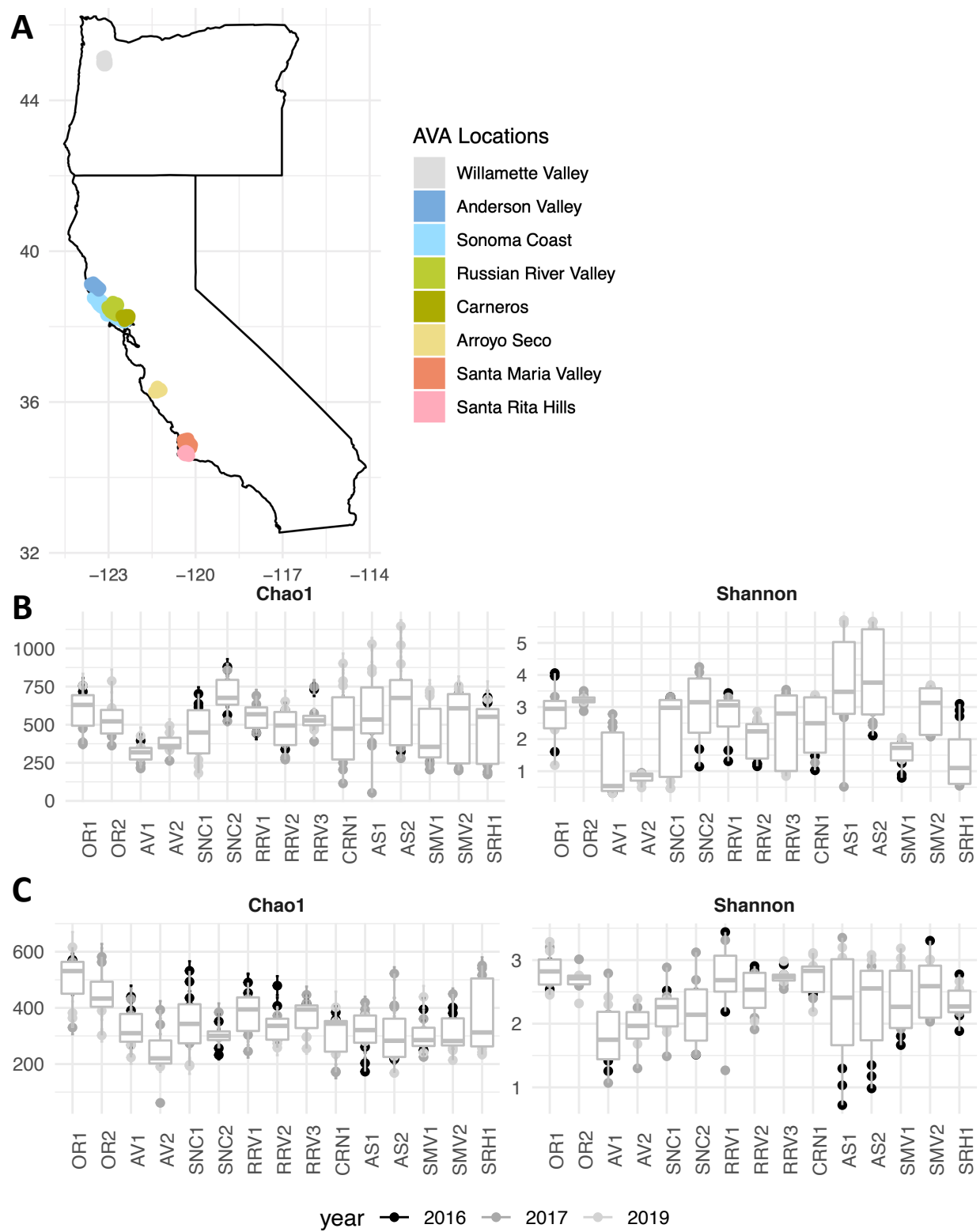

**Figure S1**

Supplement: FIG S1 [file msystems.00033-21-sf001.pdf]

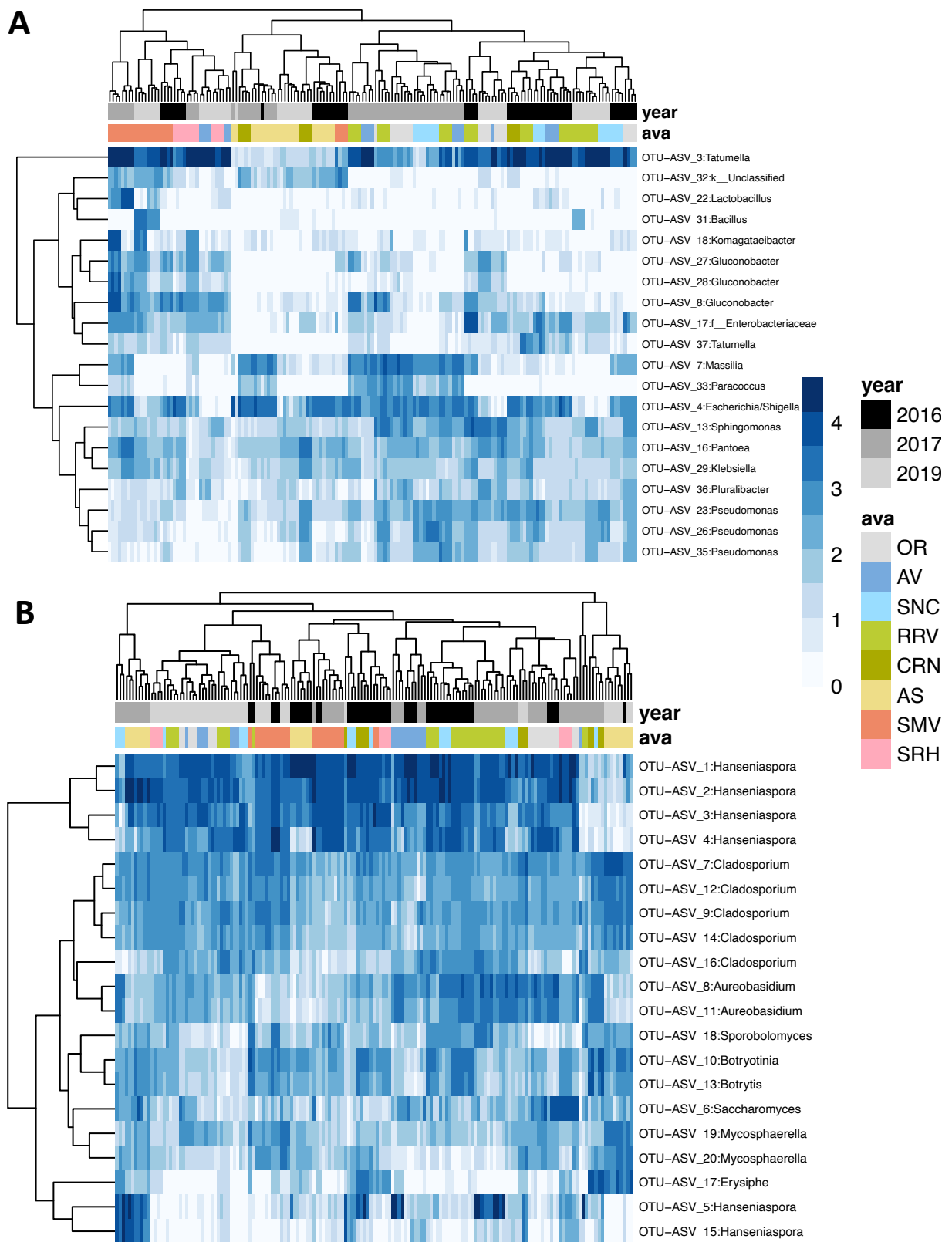

**Figure S2**

Supplement: FIG S2 [file msystems.00033-21-sf002.pdf]

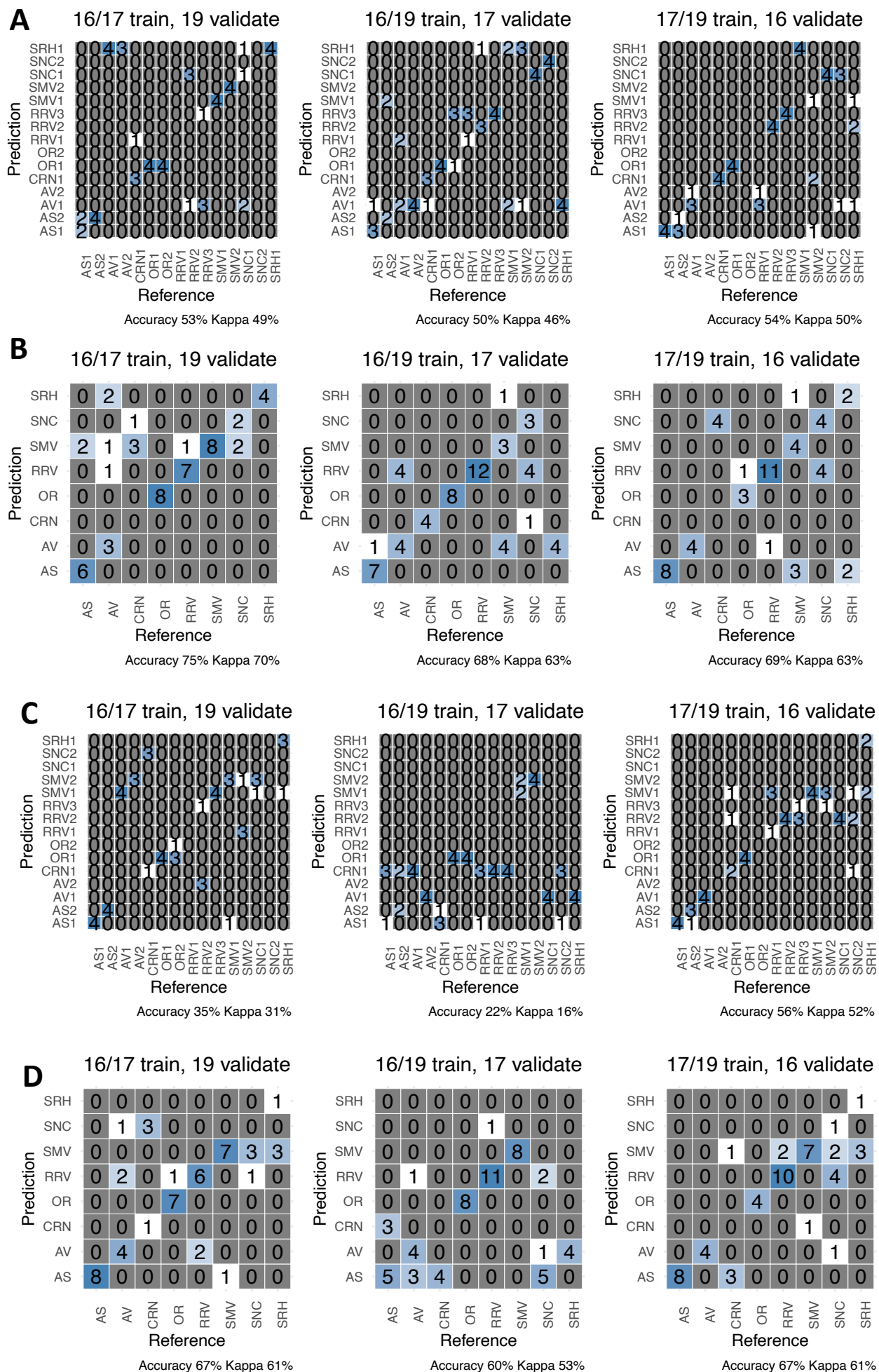

**Figure S3**

Supplement: FIG S3 [file msystems.00033-21-sf003.pdf]

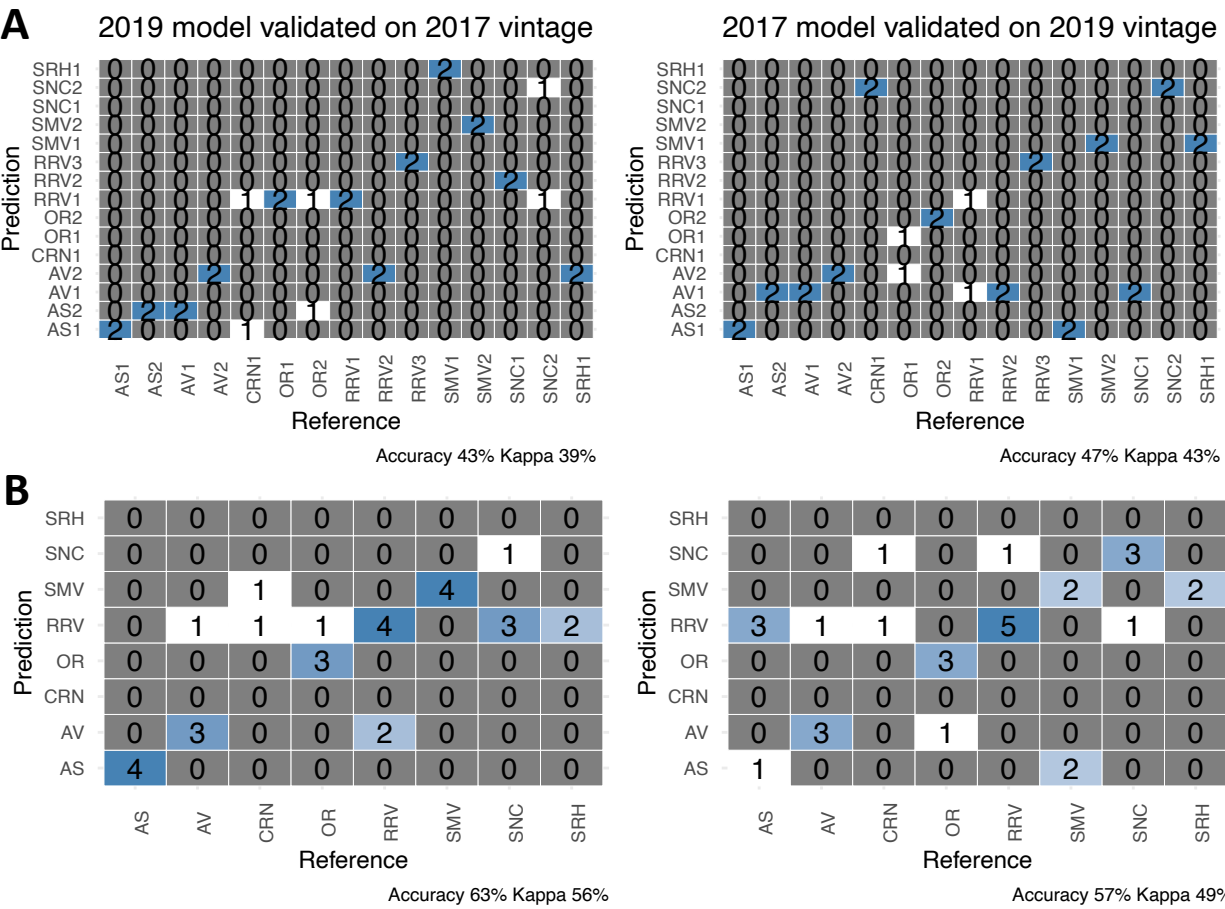

Figure S4

Supplement: FIG S4 [file msystems.00033-21-sf004.pdf]

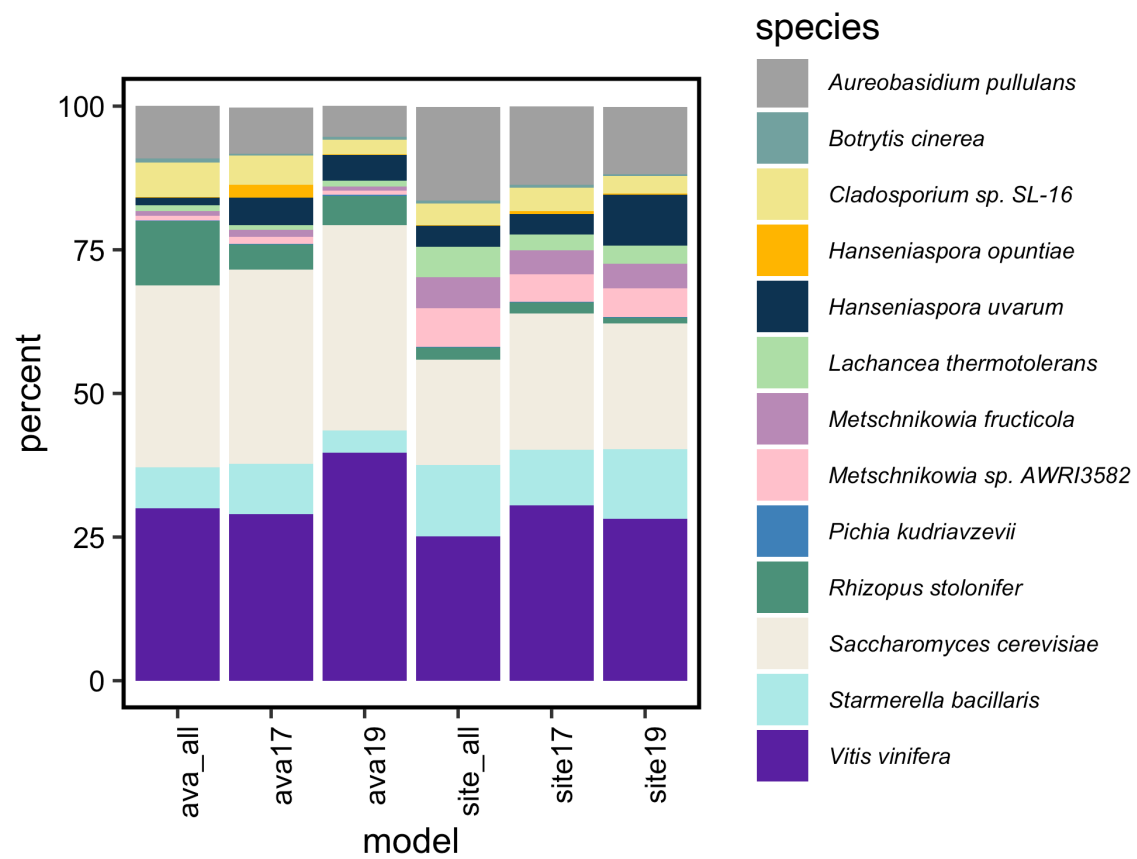

Figure S5

Supplement: FIG S5 [file msystems.00033-21-sf005.pdf]

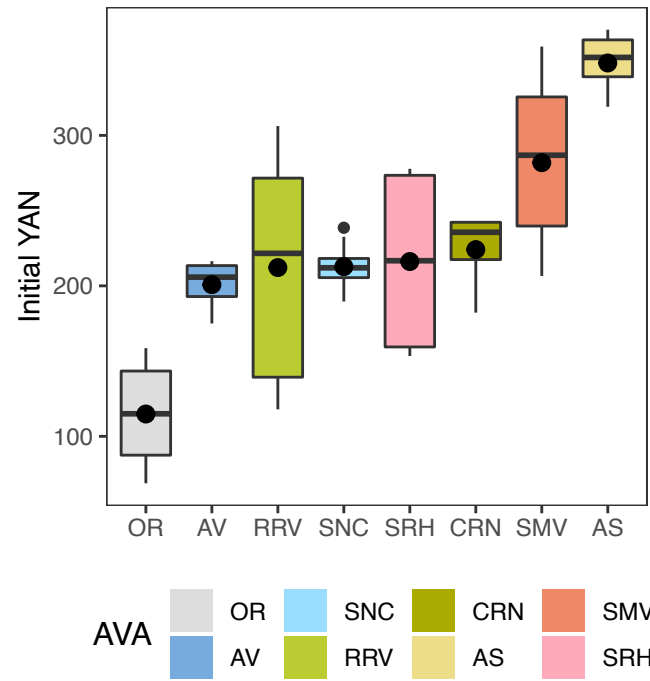

Figure S6

Supplement: FIG S6 [file msystems.00033-21-sf006.pdf]
